# Induction of IFNT-Stimulated Genes by Conceptus-Derived Exosomes during the Attachment Period

**DOI:** 10.1371/journal.pone.0158278

**Published:** 2016-06-28

**Authors:** Keigo Nakamura, Kazuya Kusama, Rulan Bai, Toshihiro Sakurai, Kazuto Isuzugawa, James D. Godkin, Yoshihito Suda, Kazuhiko Imakawa

**Affiliations:** 1 Animal Resource Science Center, Graduate School of Agricultural and Life Sciences, The University of Tokyo, Ibaraki, Japan; 2 Laboratory of Theriogenology and Animal Breeding, Graduate School of Agricultural and Life Sciences, The University of Tokyo, Tokyo, Japan; 3 Department of Occupational and Environmental Health, Faculty of Pharmaceutical Sciences, Tokyo University of Science, Chiba, Japan; 4 Department of Kampo Pharmacy, Yokohama University of Pharmacy, Kanagawa, Japan; 5 Department of Animal Sciences, The University of Tennessee, Knoxville, TN, United States of America; 6 Department of Food, Agriculture and Environment, Miyagi University, Miyagi, Japan; University of Quebec at Trois-Rivieres, CANADA

## Abstract

Biochemical and/or physical communication between the conceptus and the uterine endometrium is required for conceptus implantation to the maternal endometrium, leading to placentation and the establishment of pregnancy. We previously reported that *in vitro* co-culture system with bovine trophoblast CT-1 cells, primary uterine endometrial epithelial cells (EECs), and uterine flushings (UFs) mimics *in vivo* conceptus attachment process. To identify molecules in UFs responsible for this change, we first characterized protein contents of UFs from day 17 cyclic (C17) and pregnant (P17) ewes through the use of two dimensional-Polyacrylamide Gel Electrophoresis (2D-PAGE), followed by Liquid Chromatography-tandem Mass Spectrometry (LC-MS/MS) analysis. These analyses identified 266 proteins specific for P17 UFs, from which 172 proteins were identified as exosomal proteins. Among 172 exosomal proteins, 8 proteins that had been identified as exosomal proteins were chosen for further analysis, including macrophage-capping protein (CAPG), aldo-keto reductase family 1, member B1 protein (AKR1B1), bcl-2-like protein 15 (BCL2L15), carbonic anhydrase 2 (CA2), isocitrate dehydrogenase 2 (IDH2), eukaryotic translation elongation factor 2 (EEF2), moesin (MSN), and ezrin (EZR). CAPG and AKR1B1 were again confirmed in P15 and P17 UFs, and more importantly CAPG and AKR1B1, mRNA and protein, were found only in P15 and P17 conceptuses. Moreover, exosomes were isolated from C15, C17, P15, or P17 UFs. Only P15 and P17 exosomes, originated from the conceptus, contained interferon tau (IFNT) as well as CAPG and AKR1B1, and up-regulated *STAT1*, *STAT2*, *MX1*, *MX2*, *BST2*, and *ISG15* transcripts in EECs. These observations indicate that in addition to endometrial derived exosomes previously described, conceptus-derived exosomes are present in UFs and could function to modify endometrial response. These results suggest that exosomes secreted from conceptuses as well as endometria are involved in cell to cell interactions for conceptus implantation to the maternal endometrium.

## Introduction

Numerous studies have been conducted to elucidate molecular mechanisms by which conceptuses of ruminants attach to uterine epithelial cells and form the placenta. It is well documented that efficient biochemical communication between the conceptus and the uterine endometrium is required for conceptus implantation and placentation, leading to the establishment of pregnancy.

On day 8 of ovine pregnancy, the blastocyst hatches from the zona pellucida and begins to secrete a cytokine, interferon tau (IFNT), which peaks on day 16 and declines soon after the conceptus attaches to the uterine epithelium. It has been shown that along with maternal progesterone secretion, IFNT regulates many endometrial gene expressions, including chemokine (C-X-C motif) ligand 10 (CXCL10) and galactoside-binding, soluble, 15 (LGALS15) [1–3]. These proteins are required for conceptus attachment to the uterine epithelium. Despite the data extensively accumulated, low pregnancy rate has not been improved, resulting from early embryonic losses. These observations suggest that a factor or a mechanism, which, along with progesterone and/or IFNT, plays a major role in pregnancy establishment, has not been well-characterized.

Recently, exosomes have gained much attention because they have been implicated in various events such as cancer growth and/or metastasis. In fact, cancer cells uptake exosomes to protect them from genotoxic stress-induced cell death [4]. Bone marrow mesenchymal stromal cells from multiple myeloma (MM) patients release exosomes that express increased levels of oncogenic proteins, cytokines, and adhesion molecules to facilitate the growth of MM cells [5]. Moreover, it is reported that exosomal integrins, α6β4, α6β1 and αvβ5, are associated with lung and liver metastasis [6]. Exosomes have also been identified in many body fluids including cerebrospinal fluids [7], urine [8], and blood [9] as well as uterine flushings (UFs) [10]. The presence of exosomes in ovine UFs has been demonstrated and these exosomes are thought to be involved in conceptus-endometrial interactions during the pre-attachment period [11]. It was recently shown that endometrial exosomes released into UFs act on the trophectoderm via the toll-like receptors family to induce the secretion of IFNT during the pre-attachment phase of pregnancy [12]. These studies focused on the functions of endometrial exosomes on the conceptuses; however, the effects of conceptus exosomes on the endometrium have not been characterized.

Our laboratory has established an *in vitro* co-culture system with bovine trophoblast CT-1 cells and primary uterine endometrial epithelial cells (EECs) to study conceptus attachment processes; however, this co-culture system requires UFs from pregnant ewes to mimic the *in vivo* attachment process [13, 14]. Compared to UFs from cyclic animals, UFs recovered from pregnant animals during the attachment period dramatically changed various gene expressions in CT-1 and EECs. These results suggest that UF components during the conceptus attachment period, including various cytokines and/or exosomes, are essential for biochemical and/or physical interactions between the conceptus and the endometrium. It should be noted that the source/origin of exosomes in UFs could be conceptuses and/or endometrium.

We therefore hypothesized that the UFs during conceptus attachment period contain important molecules of conceptus and/or endometrial origins, which may regulate the uterine environment, facilitating conceptus attachment to the uterine epithelium. To examine this hypothesis, we first characterized the protein content of UFs from day 17 cyclic and pregnant ewes using two dimensional-Polyacrylamide Gel Electrophoresis (2D-PAGE) and Liquid Chromatography-tandem Mass Spectrometry (LC-MS/MS). From the results of LC-MS/MS study, we then used *in silico* analysis to identify exosomal proteins and further investigated the presence and potential function of conceptus exosomes in UFs during the attachment period.

## Materials and Methods

### Collection of Ovine Conceptuses/Endometrial Tissues, Uterine Fixation, and Uterine Flushing Preparation

All animal maintenance, care, breeding and surgical procedures were reviewed and approved by the University of Tennessee Institutional Animal Care and Use Committee (IACUC). Whiteface crossbred ewes were maintained at the farm of the University of Tennessee (UT), Knoxville, TN, USA and animal care, estrous synchronization procedures, and tissue collections were performed in accordance with the guidelines set forth by IACUC [15]. Following hysterectomy was performed in the large animal surgical suite of the Johnson Research and Teaching Unit of the University on days 15, and 17 (day 0 = day of estrus), uteri from days 15 and 17 cyclic and pregnant animals (C15, C17, P15, and P17, respectively; n = 3 each day) were flushed with 20 ml sterile PBS (pH 7.2), from which approximately 19 ml uterine flushings (UFs) including elongated conceptuses were collected. UFs were then centrifuged at 1,000 rpm for 5 min, and UFs and conceptuses were collected separately. Endometrial tissues were sampled from the uterine horns ipsilateral to the corpus luteum. All samples were frozen immediately in liquid nitrogen, and were transferred to the Laboratory of Animal Breeding at The University of Tokyo.

For uterine fixation, uteri from pregnant ewes on day 17 of gestation (n = 3) were removed and subjected to whole uterus fixation immediately after slaughter [16, 17]. Fixed, whole uteri were serially dissected into proximal to distal uterine segments, and each section was then paraffin-embedded. These paraffin-embedded tissue samples had been transported to the Laboratory of Animal Breeding at The University of Tokyo. These fixed-tissue blocks were sectioned (5 μm) and evaluated for the presence of conceptus microscopically after hematoxylin-eosin staining [16–18].

### Isolation of Exosomes from Uterine Flushings

Exosomes were isolated from 1 ml of C15, C17, P15 and P17 filtered UFs (n = 3) by adding 200 μl exosome precipitation solution (Exo-Quick-TC, System Biosciences, Mountain View, CA). The UFs with Exo-Quick-TC were incubated overnight at 4°C and then centrifuged at 1,500 x g for 30 min at 4°C to pellet exosomes. Exosomes were suspended and their protein concentration adjusted in PBS (1 μg/μl) for transmission electron microscopy analysis, nanoparticle tracking analysis, and *in vitro* culture experimentation or in mammalian protein extraction reagent (M-PER, Thermo Fisher Scientific, Inc., Waltham, MA) for western blot analysis.

### Cell Preparation and Culture Conditions

EECs were isolated and cultured as previously described [19]. In brief, uteri of healthy Holstein cows were obtained from a local abattoir in accordance with protocols approved by the local Institutional Animal Care, Use and Ethics Committee at Okayama University, Okayama, Japan. Uteri of the early luteal phase (Days 2–5) were excised and immediately transported to the laboratory. To detach EECs, the uterine lumen was trypsinized (0.3% w/v), from which EECs were isolated [19]. The isolated EECs were cultured on collagen type IA-coated six-well plates in Dulbecco-modified Eagle medium/F12 (DMEM/F12) (1:1) medium (Wako Pure Chemical Industries, Ltd., Osaka, Japan) supplemented with 10% (v/v) newborn calf serum (Invitrogen, Tokyo, Japan), 2 mM glutamine (Invitrogen), and antibiotic/antimycotic solution (Invitrogen) at 37°C under 5% CO_2_ in humidified air. EECs were used within four passages to avoid changes in cell characteristics, specifically down-regulation of steroid receptor expression [13]. In the *in vitro* cultures, EECs (1 × 10^5^ cells/well) placed onto collagen type IA-coated six-well dishes were incubated with 10 μg proteins from P17 UFs, or C15, C17, P15, or P17 exosomes in serum-free DMEM/F12 for 48 h, all of which treatments were similar to those described previously [20].

### Transmission Electron Microscopy

The exosomes in PBS were placed on carbon-film grids for 2 min, from which excess PBS was removed by touching one end of the grid with the filter paper. After grids were partially dried, the staining solution, 2% uranyl acetate in water, was added to grids and allowed to settle for 2 min. The excess liquid was blotted off by filter paper, and grids were allowed to dry overnight at room temperature. Grids were analyzed by using a HITACHI H-7600 Transmission Electron Microscopy (TEM, Hitachi High-Technologies Corporation, Tokyo, Japan), of which analysis was carried out at Hanaichi UltraStracture Research, Inc., Aichi, Japan.

### Nanoparticle Tracking Analysis

Nanoparticle tracking analysis of exosomes, isolated from P17 UF and suspended in PBS, was performed using NanoSight NS300 (NanoSight Ltd, Amesbury, UK) instrument with a 488 nm laser and a complementary metal-oxide-semiconductor (CMOS) camera (Andor Technology, Belfast, UK) and NanoSight NTA 3.2 software calibrated with 100 nm polystyrene beads (Thermo Fisher Scientific, Inc.). Particle suspensions were diluted with PBS to adjust a concentration of 2–6 x 10^8^ particles per milliliter. Videos were recorded for 30 seconds during which the nanoparticle tracking analysis software (NanoSight Ltd) tracked each visible particle. The Stokes-Einstein equation was employed to determine the size distribution and number of particles (concentration) within the sample.

### 2D-PAGE

10 μl of C17 and P17 UFs (1 μg/μl) were dissolved in 90 μl urea buffer (0.06 M Tris hydroxymethyl aminomethane, 1 M thiourea, 6 M urea, 3% CHAPS, 1% Triton X-100) and centrifuged at 15,000 × g for 30 min at 4°C. Supernatant was recovered and protein concentrations were measured using Benchmark Plus microplate spectrophotometer (Bio-Rad Laboratories, Inc., Hercules, CA).

After measuring the protein concentrations, UFs were mixed with 1 M acrylamide solution and were separated in agar gel (pH range: 3–10) (ATTO, Tokyo, Japan) and 5–20% SDS-polyacrylamide gradient gel (ATTO) according to the protocol provided by the manufacturer. After 2D-PAGE, the gel was stained with SYPRO Ruby (Thermo Fisher Scientific, Inc.) overnight and washed with distilled water. The images were then captured using an LAS-3000 camera (FUJIFILM, Tokyo, Japan).

### Analysis by LC-MS/MS

Proteins especially up-regulated in P17 UFs were identified by LC-MS/MS. After SDS-PAGE, the spots of these proteins were cut out and treated with trypsin. The tryptic digest was directly analyzed by nanoscale HPLC (MAGIC 2002, Michrom BioResources Inc., Auburn, CA) on a C18 column (0.1 × 50 mm, Michrom BioResources Inc.) coupled to a tandem mass spectrometer (Q-Tof2, Micromass, London, UK) equipped with a nanoelectrospray ionization source. Tandem mass spectra were analyzed using Mascot, which allows for the correlation of experimental data with theoretical spectra generated from known protein sequences. The data were used to search a compiled protein database, NCBInr, which is publicly available (http://www.ncbi.nlm.nih.gov/).

### RNA Extraction and Quantitative RT-PCR

Using the ISOGEN reagent (Nippon Gene, Tokyo, Japan), total RNAs were extracted from days 15 and 17 conceptuses and endometria and cultured EECs according to the manufacturer’s protocol. For quantitative real-time PCR (qPCR) analyses, isolated RNA (total 250 ng) was reverse-transcribed to cDNA using ReverTra Ace qRNA RT Kit (Toyobo, Osaka, Japan), and the resulting cDNA (RT template) was stored at 4°C until use [21].

Reverse-transcribed cDNA was subjected to qPCR amplification using Thunderbird SYBR qPCR Mix Kit (Toyobo) with 0.3 μM of the oligonucleotide primers listed in S1 Table, and qPCR amplification was carried out on an Applied Biosystems STEP One Plus real-time PCR System (Applied Biosystems, Foster City, CA) [22]. Amplification efficiencies of each target gene and two reference genes, bovine beta-action (*ACTB*) and glyceraldehyde 3-phosphate dehydrogenase (*GAPDH*), were examined through their calibration curves and found to be comparable [14]. The thermal profile for qPCR consisted of 40 cycles at 95°C for 10 sec and annealing and extension at 60°C for 45 sec. Average threshold (Ct) values for each target were determined by Sequence Detection System software v2.2 (Applied Biosystems). Each run was completed with melting curve analysis to confirm the specificity of amplification and the absence of primer dimer.

### Western Blot Analysis

To determine expressions of macrophage-capping protein (CAPG), aldo-keto reductase family 1, member B1 protein (AKR1B1), tetraspanin protein 63 (CD63), heat shock protein 70 (HSP70), or IFNT protein in frozen samples, conceptuses or endometrial tissues were prepared in lysis buffer (50 mM Tris-HCl, 150 mM NaCl, 1 mM ethylenediaminetetraacetic acid (EDTA), 1% Triton X-100, 0.1% sodium dodecyl sulfate, 1 mM Na_3_VO_4_, and 50 mM NaF). Tissue lysates, UFs or exosomes prepared in M-PER (10 μg/lane) were separated through 12.5% SDS-PAGE and were then transferred onto polyvinylidene difluoride (PVDF) membranes (n = 3) (Millipore, Milford, MA). After blocking with Block Ace reagent (DS Pharma Biomedical, Osaka, Japan), membranes were incubated with a goat polyclonal anti-human CAPG polyclonal antibody(2 μg/ml, sc-33084, Santa Cruz Biotechnology, Inc., Dallas, TX), a rabbit polyclonal anti-human AKR1B1 antibody (1 μg/ml, sc-33219, Santa Cruz Biotechnology, Inc.), rabbit monoclonal anti-bovine IFNT antibody (1:1000 dilution, Eurofins Genomics, Inc., Ebersberg, Germany), rabbit monoclonal anti-human ACTB antibody (for internal control, 1:1000, ab1801, Abcam, Tokyo, Japan), rabbit polyclonal anti-human CD63 antibody (for exosome marker, 0.25 μg/ml, EXOAB-CD63A-1, System Biosciences), or rabbit polyclonal anti-human HSP70 antibody (for exosome marker, 0.25 μg/ml, EXOAB-HSP70A-1, System Biosciences). Immunoreactive bands were detected using enhanced chemiluminescence (Millipore) after incubation with horseradish peroxidase-labeled SAP solution (APRO life Science, Inc., Tokushima, Japan).

### Immunohistochemistry

Immunohistochemical analyses were performed on 5 μm paraffin sections of day 17 uterine tissue blocks. Paraffin sections were rehydrated and boiled for 20 min in 10 mM citrate buffer (pH 6.0), and endogenous peroxidase was quenched by immersing in 0.3% (v/v) hydrogen peroxide/methanol, as described previously [17, 18]. A streptavidin/biotin blocking kit (Vector Laboratories, Burlingame, CA) was used to block endogenous biotin according to the manufacturer’s instructions. After 30 min incubation with 10% normal goat or donkey serum, the sections were incubated at 4°C overnight with a goat anti-human CAPG polyclonal antibody (2 μg/ml, sc-33084, Santa Cruz Biotechnology, Inc.), or a rabbit anti-human AKR1B1 polyclonal antibody (4 μg/ml, sc-33219, Santa Cruz Biotechnology, Inc.), or the respective negative controls; normal goat IgG (10 μg/ml, sc-2028, Santa Cruz Biotechnology, Inc.) or normal rabbit IgG (10 μg/ml, sc-2027, Santa Cruz Biotechnology, Inc.). Subsequently, the sections were incubated at room temperature for 1 h with either a donkey anti-goat IgG-HRP (1 μg/ml, sc-2020, Santa Cruz Biotechnology, Inc) or a goat anti-rabbit IgG biotin conjugate (1:400 dilution, B8895, Sigma-Aldrich, St. Louis, MO). The immunoreactivity was visualized by means of avidin-peroxidase (1:400 dilution, E2886, Sigma-Aldrich) and AEC substrate kits (Invitrogen) according to the manufacturer’s instructions and then examined under light microscope (BX-51, Olympus, Tokyo, Japan).

### Statistical Analysis

Data are expressed as the mean ± SEM. Significance was assessed using t-test or the Tukey-Kramer test. A *P*-value < 0.05 was considered statistically significant.

## Results

### UFs from Day 17 Pregnant Ewes Contain Up-Regulated Proteins

**Fig 1:**
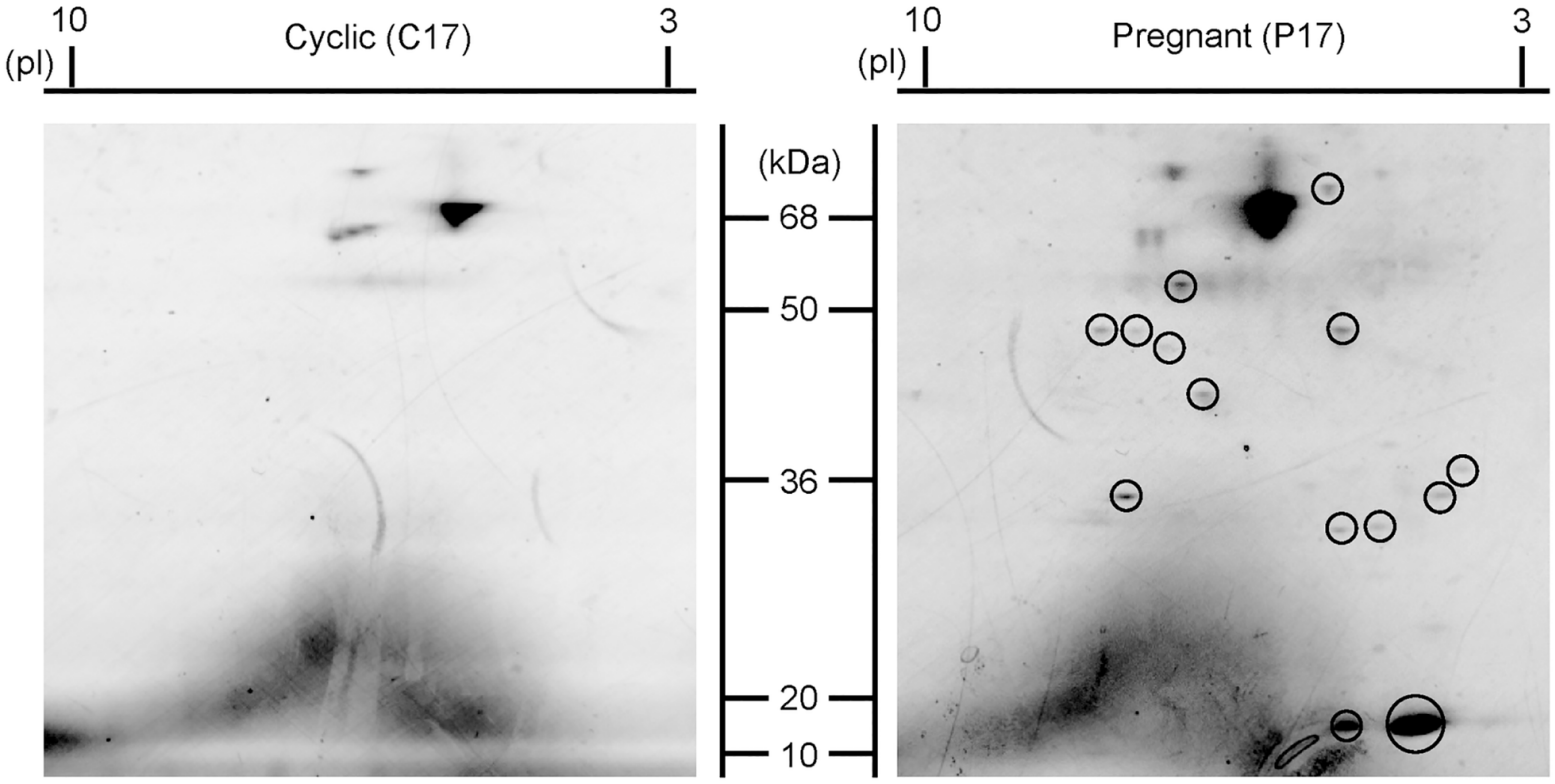
Use of 2D-PAGE to identify proteins specific to day 17 pregnant UFs. Representative SYPRO Ruby stained 2D gel images of UFs from day 17 cyclic (left) or pregnant (right) ewes. Several spots designated by circle in day 17 pregnant UFs were up-regulated compared to those in day 17 cyclic UFs, which were subjected to LC-MS/MS analysis.

Results from 2D-PAGE revealed that although similar protein migration patterns between C17 and P17 UFs were recognized, P17 UFs contain increased proteins compared to those of C17 (Fig 1). Spots especially up-regulated in P17 UFs were cut off and subjected to LC-MS/MS analysis, which revealed that a total of 266 proteins were identified from the P17 up-regulated spots (see S2 Table). These proteins were further analyzed through the use of Database for Annotation, Visualization and Integrated Discovery (DAVID) (https://david.ncifcrf.gov/) and ExoCarta (http://exocarta.ludwig.edu.au), resulting in the identification of 13 secretory proteins and 172 exosomal proteins. Based on the previous results of UF analyses [23, 24], the 8 exosomal proteins CAPG, AKR1B1, bcl-2-like protein 15 (BCL2L15), carbonic anhydrase 2 (CA2), isocitrate dehydrogenase 2 (IDH2), eukaryotic translation elongation factor 2 (EEF2), moesin (MSN), and ezrin (EZR) were selected for further analysis.

### Up-Regulation of *CAPG* and *AKR1B1* mRNAs are Specific to Day 15 and 17 Conceptuses

**Fig 2:**
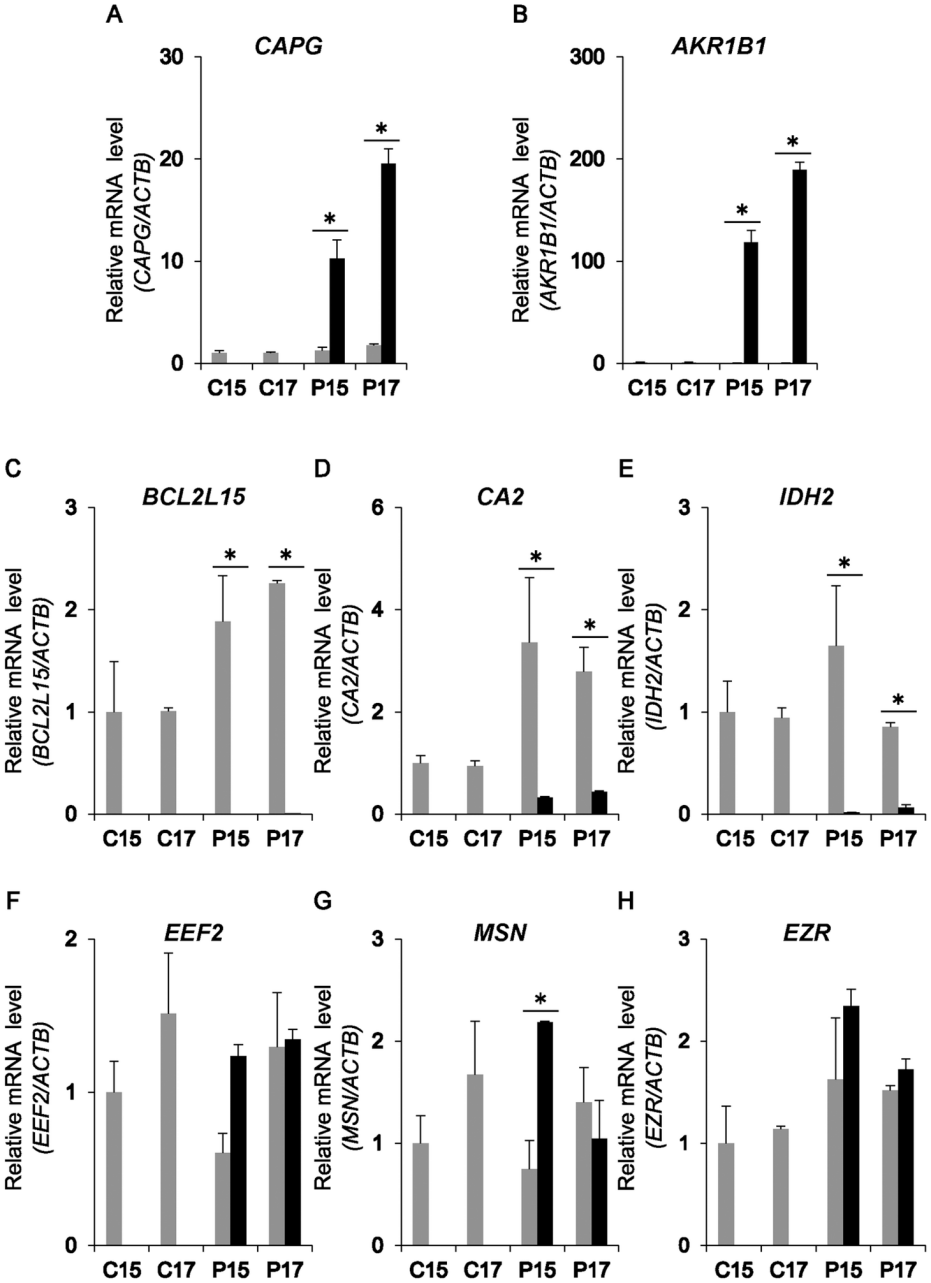
Levels of 8 exosomal transcripts in endometria and conceptuses. The relative mRNA expression of 8 exosomal transcripts, *CAPG* (A), *AKR1B1* (B), *BCL2L15* (C), *CA2* (D), *IDH2* (E), *EEF2* (F), *MSN* (G), *EZR* (H), in C15, C17, P15, or P17 endometria (grey bar) and P15 or P17 conceptuses (solid bar) (n = 3 each day). *ACTB* and *GAPDH* were used as internal controls for RNA integrity. Values represent mean ± SEM. *Statistically significant difference between conceptus and endometrium in each day (P<0.05).

To identify the source of exosomal proteins unique to P17 UFs, real-time PCR was executed to examine transcripts of *CAPG*, *AKR1B1*, *BCL2L15*, *CA2*, *IDH2*, *EEF2*, *MSN*, and *EZR* in the RNAs extracted from C15, C17, P15, and P17 endometrial tissues, and P15 and P17 conceptus tissues. *CAPG* and *AKR1B1* mRNAs were up-regulated in conceptus tissues (Fig 2A and 2B), whereas *BCL2L15*, *CA2*, and *IDH2* mRNAs were expressed in C15, C17, P15, and P17 endometrial tissues (Fig 2C, 2D and 2E). Expression of *EEF2*, *MSN*, and *EZR* transcripts was similar between endometrium and conceptus tissues (Fig 2F, 2G and 2H).

### CAPG and AKR1B1 Proteins Are Expressed by Conceptuses

**Fig 3:**
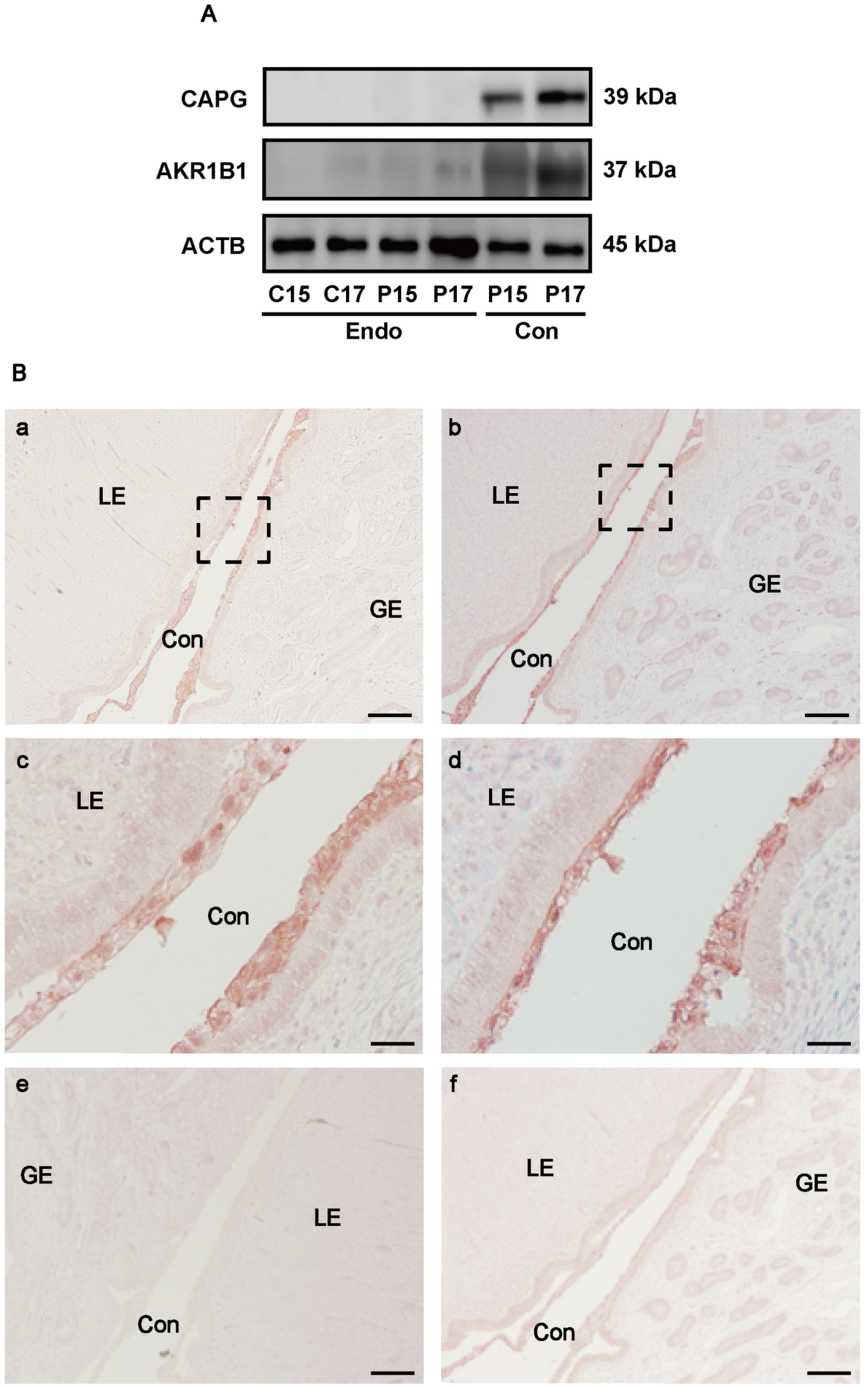
Expression and localization of CAPG and AKR1B1 proteins in the conceptuses. (A) The presence of CAPG or AKR1B1 proteins in C15, C17, P15, or P17 ovine endometria and P15 or P17 conceptuses was examined by western blot analysis. ACTB was used as an internal control. A representative data from three independent experiments containing protein samples from different animals was shown. Endo, endometrium; Con, conceptus. (B) Immunohistochemical detection of CAPG (a, c, and e) and AKR1B1 (b, d, and f) in P17 ovine uteri. Tissue sections were immunostained for CAPG using anti-CAPG antibody (a and c) or normal goat IgG (e) as a negative control. Boxed area in (a) is shown at a higher magnification (c). Tissue sections were immunostained for AKR1B1 using anti- AKR1B1 antibody (b and d) or normal rabbit IgG (f) as a negative control. Boxed area in (b) is shown at a higher magnification (d). Con, conceptus; LE, luminal epithelium; GE, glandular epithelium. Scale bar = 250 μm (a, b, e, and f), or 50μm (c and d), respectively.

Since *CAPG and AKR1B1* mRNAs were found only in P15 and P17 conceptus tissues, the expression and localizations of these proteins were further examined in C15, C17, P15, and P17 endometrial tissues, and P15 and P17 conceptus tissues. Western blot analysis revealed that similar to *CAPG* and *AKR1B1* transcripts, CAPG protein was specifically expressed in P15 and P17 conceptuses. Minute expression of AKR1B1 protein was found in P15 and P17 endometrium, but definitive expression was found in P15 and P17 conceptuses (Fig 3A). To confirm the localizations of CAPG and AKR1B1 proteins associated with its transcript expression *in utero*, immunohistochemical analysis was carried out with paraffin sections from P17 uterine tissues. Consistent with the results from western blot analysis, CAPG and AKR1B1 proteins were localized to the conceptuses, but minute expression of AKR1B1 was also found in the endometrial glandular epithelia (Fig 3B).

### Exosomes Are Present in Cyclic and Pregnant UFs, but CAPG and AKR1B1 Proteins Are Found in Exosomes from P15 and P17 UFs

**Fig 4:**
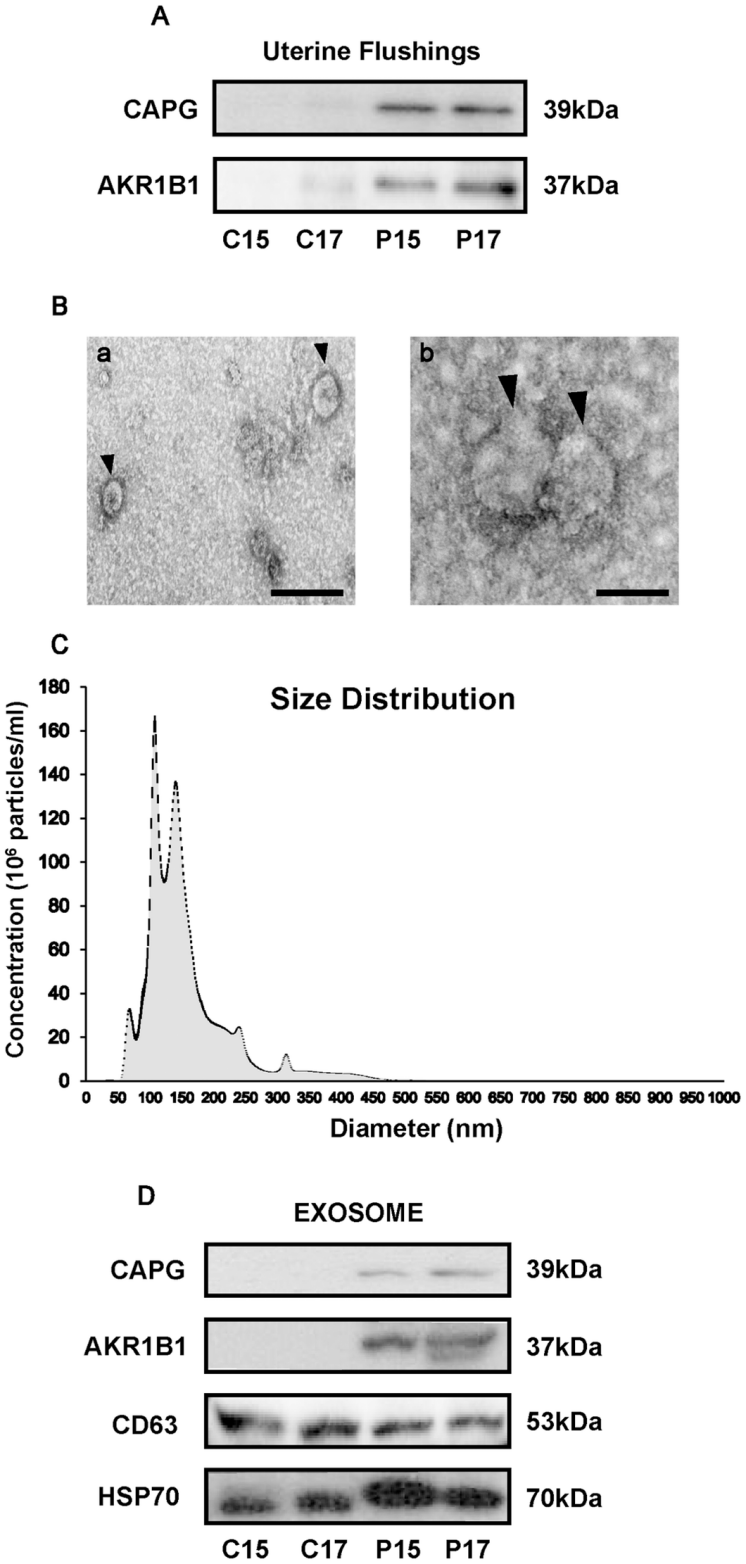
Identification of exosomal CAPG and AKR1B1 in days 15 and 17 pregnant UFs. (A) The presence of CAPG and AKR1B1 in C15, C17, P15, or P17 UFs (10 μg each) was examined by western blot analysis (n = 3 each day). (B) Transmission electron microscopy analysis revealed the presence of approximately 150 nm vesicles in UFs, consistent with exosomes. Scale bar = 200 nm (a) or 100 nm (b). (C) Nanoparticle tracking analysis (n = 3, triplet analysis in each sample) of P17 UF revealed that a range of exosomal size is 50 to 150 nm. Gray area in (C) represents an average from three samples, whereas black area represents SEM. (D) Western blot analysis showed the presence of CD63 and HSP70 in exosomes isolated from C15, C17, P15, or P17 UFs, and the presence of CAPG and AKR1B1 in exosomes isolated from days 15 and 17 pregnant UFs (n = 3 each day). In (A), (B), and (D), a representative one is shown.

Western blot analysis revealed that CAPG and AKR1B1 proteins were present in P15 and P17 UFs (Fig 4A). Transmission electron microscopy detected vesicles of approximately 150 nm in diameter in the isolated pellets (Fig 4B), and separated exosome preparations from P17 UF were subjected to nanoparticle tracking analysis, revealing a mean of 131.8 nm, standard deviation of 61.9 nm, mode of 102.1 nm, and a range of 50 to 200 nm (Fig 4C). While exosomal protein markers CD63 and HSP70 were positive in all isolated pellets, CAPG and AKR1B1 proteins were only identified in exosomes isolated from P15 and P17 UFs (Fig 4D). These results indicated that exosomes containing CAPG and AKR1B1 were secreted from days 15 and 17 conceptuses during the attachment period.

### Exosomes Isolated from P15 and P17 UFs Contain IFNT, Which Up-Regulates Interferon Stimulated Genes (ISGs) in EECs

**Fig 5:**
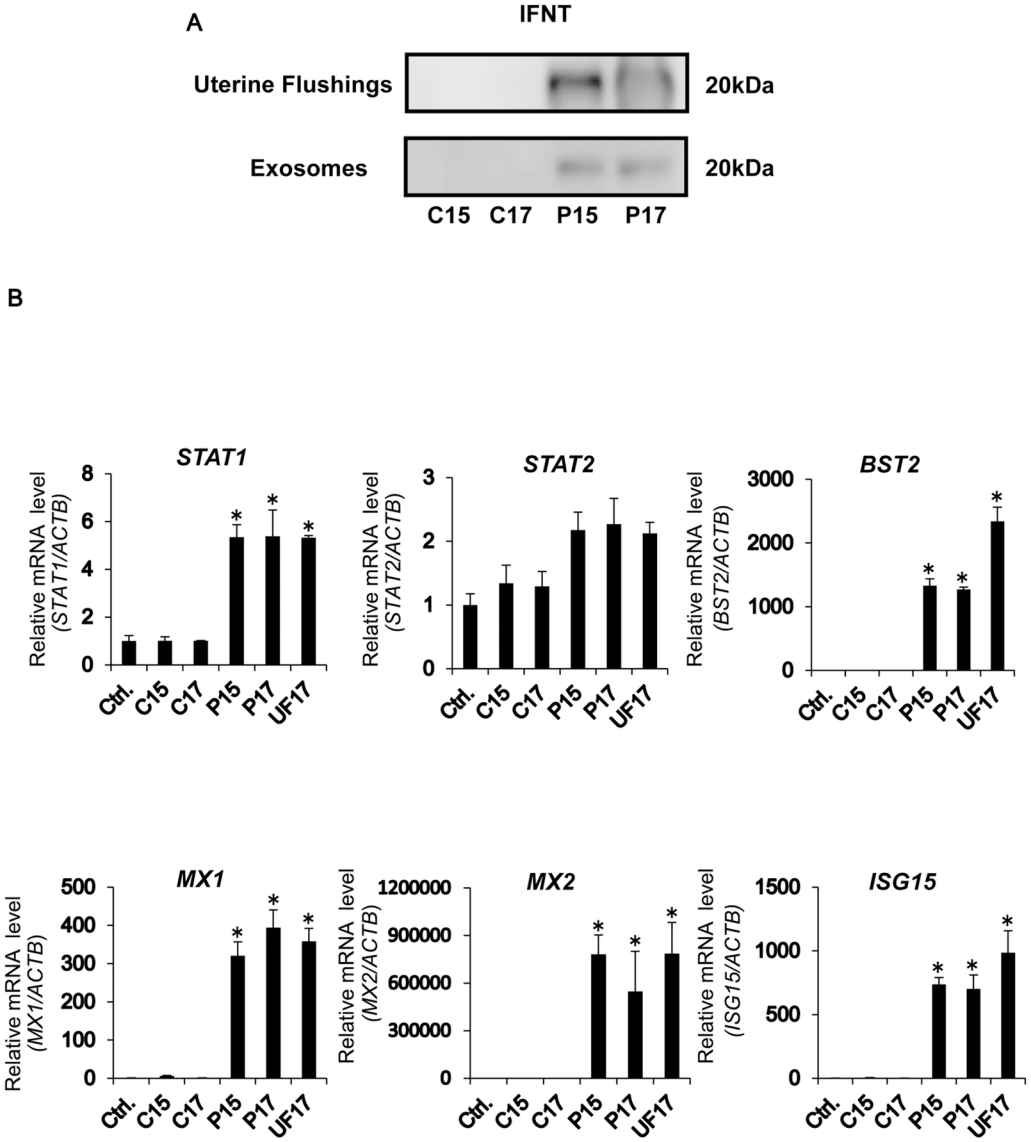
Presence of IFNT in UFs and exosomes from days 15 and 17 pregnant ewes and the effect of those exosomes on EECs. (A) The presence of IFNT in C15, C17, P15, or P17 UFs and exosomes was examined by western blot analysis (n = 3 each day), and IFNT was detected in P15 and P17 UFs. A representative of three independent experiments is shown. (B) Effects of exosomes on EECs. EECs were treated with or without C15, C17, P15, or P17 exosome (10 μg each), or P17 UF (10 μg proteins) for 48 h. RNA was extracted from EECs and subjected to real-time PCR analysis for *STAT1*, *STAT2*, *BST2*, *MX1*, *MX2*, and *ISG15* transcript levels. *ACTB* and *GAPDH* mRNA were used as internal controls for RNA integrity. Values were from three independent experiments, each containing duplicates. Values represent mean ± SEM. *Statistically significant difference in mRNA levels vs. control (Ctrl) without exosomes or UFs treatment (P<0.05).

Consistent with the study by Ruiz-González et al. [12], P15 and P17 UFs contained IFNT, and exosomes isolated from P15 and P17 UFs contained IFNT (Fig 5A). To study potential functions of these exosomes, UFs or isolated exosomes were added to the cultured EECs, from which RNA was extracted and subjected to qPCR analysis to determine changes in ISGs, *STAT1*, *STAT2*, *MX1*, *MX2*, *BST2*, and *ISG15* transcripts. The results revealed that ISG transcripts in EECs treated with P17 UFs, and P15 or P17 exosomes were up-regulated (Fig 5B). These results indicated that up-regulation of ISGs expression in EECs could result from the IFNT-containing exosomes released from the conceptuses.

## Discussion

The results from this study provide the evidence that conceptus-derived exosomes are present in P15 and P17 UFs and could potentially function to modify endometrial response during the attachment period. Compared to C17, proteins unique to the existence of conceptuses are predominantly present in P17 UFs, and many of them are associated with extracellular vesicular exosomes and extracellular region/matrix. In addition, CAPG and AKR1B1 predominantly expressed by conceptuses are present in isolated exosomes during the attachment period. Moreover, ISGs mRNA expressions in EECs are up-regulated in response to the exosomes containing IFNT, suggesting that exosomes secreted from conceptuses are involved in the generation of uterine environment required for pregnancy establishment.

Results of 2D-PAGE followed by LC-MS/MS analysis revealed that P17 UFs contained 267 specific proteins, from which DAVID and ExoCarta revealed 13 secretory proteins and 172 exosomal proteins, respectively. These observations, together with the reports that exosomes are present in UFs during the pre-attachment period in ewe [11, 12], indicate that exosomes present in pregnant UFs during the attachment period could be responsible for the regulation of genes in bovine trophoblast CT1 cells and EECs in our previous studies [13, 14].

Conceptus and cancer cells possess various characteristics in common. Both, for instance, undergo an epithelial-mesenchymal transition (EMT) to acquire adhesion and/or invasive competence [22, 25]. In addition, hypoxia promotes cancer cells and conceptus cells for proliferation and angiogenesis [26, 27]. Furthermore, a number of recent evidence indicates that cancer cell-derived exosomes mediate the interaction between cancer cells and their neighboring cells, which play a role in cancer development and invasion [28–30]. These findings support a notion that conceptuses produce exosomes to facilitate conceptus-uterine interaction in a manner similar to that whereby cancer cells secrete exosomes to serve as mediators with target cells. Based on this idea, exosomal proteins derived from conceptuses during the attachment period were re-evaluated, and 8 exosomal proteins, CAPG, AKR1B1, BCL2L15, CA2, IDH2, EEF2, MSN, EZR, were selected as more reliable exosomal proteins. Selection of the eight exosomal proteins, out of 172 classified by ExoCarta, was based upon the observations of UFs from pregnant animals as described by other investigators [23, 24]. In addition to the endometrium derived exosomes, CAPG and AKR1B1 expressions found from this study suggest that exosomes derived from conceptuses are present in UFs during the attachment period.

In recent years, several proteomic studies demonstrated that CAPG is overexpressed in various types of cancer [31–33], which enhances cellular motility/chemotaxis [34], and are associated with increased invasion into collagen type I or chick heart fragments [35]. Moreover, AKR1B1 has been shown as a molecule associated with EMT [36] and angiogenesis [37]. Because conceptus and cancer cells possess various characteristics in common such as EMT and angiogenesis, it is considered that CAPG and AKR1B1 included in conceptus-derived exosomes could be involved in the conceptus attachment similar to those demonstrated in cancer adhesion and/or invasion. However, the elucidation of exact function exhibited by CAPG and AKR1B1 in exosomes, particularly within the context of conceptus attachment to the uterine epithelium requires further experimentation.

Nanoparticles isolated from UFs were confirmed to be exosomes through their size and morphology as seen by TEM and nanoparticle tracking analysis. In addition, CD63 and HSP70 were present in all exosomes isolated from C15, C17, P15, or P17 UFs, indicating that exosomes are present within the uterine lumen in cyclic ewes as well as pregnant ewes [38, 39]. However, CAPG and AKR1B1, which were mainly expressed in P15 and P17 conceptuses, were present only in exosomes isolated from P15 and P17 UFs during the attachment period. This study supports the hypothesis that exosomes containing CAPG and AKR1B1, secreted by conceptuses, are released into the uterine lumen during the attachment period. In the previous analysis by other investigators, 30 conceptus-derived proteins were identified in UFs during the pre-attachment period and were thought to facilitate biochemical and/or physical interactions between the conceptus and the endometrium via a micro-vesicular transport mechanism [24]. This and our results suggest that exosomes released from the conceptus could play a role in carrying these proteins.

IFNT has been considered to be the main trophoblast factor which acts on uterine endometrium and abrogates luteolytic mechanisms [40], resulting in the continued production of progesterone. Recently, however, extra-uterine or endocrine effects of IFNT have been noted. For instance, MX gene expression predominantly increases in peripheral blood cells (PBMC) in response to initial IFNT signaling in pregnant ewes [41]. Compared with the corpus luteum (CL) in non-pregnant ewes, the CL in pregnant ewes has a higher number of neutrophils, which are influenced by IFNT [42]. In addition, IFNT induces *ISG15* gene expression in jugular blood and liver tissue and several ISGs including ISG15, STAT1, and STAT2 in CL via uterine veins [43, 44]. It has been reported that blood also contains exosomes for organ-to-organ communications [45, 46]. Results from our studies together with these observations suggest that exosomes including IFNT could be involved in the regulation of ISGs in extra-uterine tissues as well. However, the exact mechanisms by which IFNT directly or indirectly functions on the extra-uterine tissues such as CL or PBMC remain unknown. Further experiment is required to clarify how trophoblast IFNT reaches CL or effects on extra-uterine tissues.

In conclusion, we propose that IFNT-containing exosomes derived from conceptus may facilitate conceptus-endometrium interaction, resulting in the generation of uterine environment required for conceptus attachment to the uterine endometrium.

## Supporting Information

S1 Table Oligonucleotide primers for real-time PCR analyses. (XLSX)

S2 Table Lists of proteins from LC-MS/MS analysis. (XLSX)

## References

[1] Nagaoka K, Sakai A, Nojima H, Suda Y, Yokomizo Y, Imakawa K, et al A chemokine, interferon (IFN)-gamma-inducible protein 10 kDa, is stimulated by IFN-tau and recruits immune cells in the ovine endometrium. Biol Reprod. 2003; 68: 1413–1421. 12606423 10.1095/biolreprod.102.008912

[2] Nagaoka K, Nojima H, Watanabe F, Chang KT, Christenson RK, Sakai S, et al Regulation of blastocyst migration, apposition, and initial adhesion by a chemokine, interferon gamma-inducible protein 10 kDa (IP-10), during early gestation. J Biol Chem. 2003; 278: 29048–29056. 12756249 10.1074/jbc.M300470200

[3] Gray CA, Adelson DL, Bazer FW, Burghardt RC, Meeusen EN, Spencer TE. Discovery and characterization of an epithelial-specific galectin in the endometrium that forms crystals in the trophectoderm. Proc Natl Acad Sci U S A. 2004; 101: 7982–7987. 15148380 10.1073/pnas.0402669101 PMC419543

[4] Khan S, Aspe JR, Asumen MG, Almaguel F, Odumosu O, Acevego-Martinez S, et al Extracellular, cell-permeable surviving inhibits apoptosis while promoting proliferative and metastatic potential. Br J Cancer. 2009; 100: 1073–1086. 10.1038/sj.bjc.6604978 19293795 PMC2669990

[5] Roccaro AM, Sacco A, Maiso P, Azab AK, Tai YT, Reagan M, et al BM mesenchymal stromal cell-derived exosomes facilitate multiple myeloma progression. J Clin Invest. 2013; 123: 1542–1555. 23454749 10.1172/JCI66517 PMC3613927

[6] Hoshino A, Costa-Silva B, Shen TL, Rodrigues G, Hashimoto A, Tesic Mark M, et al Tumour exosome integrins determine organotropic metastasis. Nature. 2015; 527: 329–335. 10.1038/nature15756 26524530 PMC4788391

[7] Street JM, Barran PE, Mackay CL, Weidt S, Balmforth C, Walsh TS, et al Idetification and proteomic profiling of exosomes in human cerebrospinal fluid. J Transl Med. 2012; 10: 5 10.1186/1479-5876-10-5 22221959 PMC3275480

[8] Prunotto M, Farina A, Lane L, Pernin A, Schifferli J, Hochstrasser DF, et al Proteomic analysis of podocyte exosome-enriched fraction from normal human urine. J Proteomics. 2013; 82: 193–229. 10.1016/j.jprot.2013.01.012 23376485

[9] Cleys ER, Halleran JL, McWhorter E, Hergenreder J, Enriquez VA, da Silveira JC, et al Identification of microRNAs in exosomes isolated from serum and umbilical cord blood, as well as placentomes of gestational day 90 pregnant sheep. Mol Reprod Dev. 2014; 81: 983–993. 10.1002/mrd.22420 25269776

[10] Ng YH, Rome S, Jalabert A, Forterre A, Singh H, Hincks CL, et al Endometrial exosomes/microvesicles in the uterine microenvironment: a new paradigm for embryo-endometrial cross talk at implantation. PLoS One. 2013; 8: e58502 10.1371/journal.pone.0058502 23516492 PMC3596344

[11] Burns G, Brooks K, Wildung M, Navakanitworakul R, Christenson LK, Spencer TE. Extracellular vesicles in luminal fluid of the ovine uterus. PLoS One. 2014; 9: e90913 10.1371/journal.pone.0090913 24614226 PMC3948691

[12] Ruiz-González I, Xu J, Wang X, Burghardt RC, Dunlap KA, Bazer FW. Exosomes, endogenous retroviruses and toll-like receptors: pregnancy recognition in ewes. Reproduction. 2015;149: 281–291. 10.1530/REP-14-0538 25526899

[13] Sakurai T, Bai H, Bai R, Arai M, Iwazawa M, Zhang J, et al Coculture system that mimics in vivo attachment processes in bovine trophoblast cells. Biol Reprod. 2012; 87: 60 10.1095/biolreprod.112.100180 22723465

[14] Bai R, Kusama K, Sakurai T, Bai H, Wang C, Zhang J, et al The Role of Endometrial Selectins and Their Ligands on Bovine Conceptus Attachment to the Uterine Epithelium During Peri-Implantation Period. Biol Reprod. 2015; 93: 46 10.1095/biolreprod.115.128652 26134867

[15] Sakurai T, Bai H, Konno T, Ideta A, Aoyagi Y, Godkin JD, et al Function of a transcription factor CDX2 beyond its trophectoderm lineage specification. Endocrinology. 2010; 151: 5873–5881. 10.1210/en.2010-0458 20962045

[16] Imakawa K, Tamura K, Lee RS, Ji Y, Kogo H, Sakai S, et al Temporal expression of type I interferon receptor in the peri-implantation ovine extra-embryonic membranes: demonstration that human IFNalpha can bind to this receptor. Endocr J. 2002; 49: 195–205. 12081239 10.1507/endocrj.49.195

[17] McGuire WJ, Imakawa K, Tamura K, Meka CS, Christenson RK. Regulation of endometrial granulocyte macrophage-colony stimulating factor (GM-CSF) in the ewe. Domest Anim Endocrinol. 2002; 23: 383–396. 12206872 10.1016/s0739-7240(02)00181-9

[18] Imakawa K, Kim MS, Matsuda-Minehata F, Ishida S, Iizuka M, Suzuki M, et al Regulation of the ovine interferon-tau gene by a blastocyst-specific transcription factor, Cdx2. Mol Reprod Dev. 2006; 73: 559–567. 16489630 10.1002/mrd.20457

[19] Skarzynski DJ, Miyamoto Y, Okuda K. Production of prostaglandin f (2alpha) by cultured bovine endometrial cells in response to tumor necrosis factor alpha: cell type specificity and intracellular mechanisms. Biol Reprod. 2000; 62: 1116–1120. 10775156 10.1095/biolreprod62.5.1116

[20] Bai R, Bai H, Kuse M, Ideta A, Aoyagi Y, Fujiwara H, et al Involvement of VCAM1 in the bovine conceptus adhesion to the uterine endometrium. Reproduction. 2014; 148: 119–127. 10.1530/REP-13-0655 24803492

[21] Bai H, Sakurai T, Kim MS, Muroi Y, Ideta A, Aoyagi Y, et al Involvement of GATA transcription factors in the regulation of endogenous bovine interferon-tau gene transcription. Mol Reprod Dev. 2009; 76: 1143–1152. 10.1002/mrd.21082 19598245

[22] Yamakoshi S, Bai R, Chaen T, Ideta A, Aoyagi Y, Sakurai T, et al Expression of mesenchymal-related genes by the bovine trophectoderm following conceptus attachment to the endometrial epithelium. Reproduction. 2012; 143: 377–387. 10.1530/REP-11-0364 22157247

[23] Koch JM, Ramadoss J, Magness RR. Proteomic profile of uterine luminal fluid from early pregnant ewes. J Proteome Res. 2010; 9: 3878–3885. 10.1021/pr100096b 20578732 PMC3124775

[24] Forde N, Bazer FW, Spencer TE, Lonergan P. 'Conceptualizing' the Endometrium: Identification of Conceptus-Derived Proteins During Early Pregnancy in Cattle. Biol Reprod. 2015; 92: 156 10.1095/biolreprod.115.129296 25947061 PMC4652614

[25] Guan X. Cancer Metastases: challenges and opportunities. Acta Pharm Sin B. 2015; 5: 402–418. 10.1016/j.apsb.2015.07.005 26579471 PMC4629446

[26] Dong J, Xu J, Wang X, Jin B. Influence of the interaction between long noncoding RNAs and hypoxia on tumorigenesis. Tumour Biol. 2015; in press. 10.1007/s13277-015-4457-0 26608368

[27] Jeong W, Bazer FW, Song G, Kim J. Expression of hypoxia-inducible factor-1 by trophectoderm cells in response to hypoxia and epidermal growth factor. Biochem Biophys Res Commun. 2015; in press. 10.1016/j.bbrc.2015.11.091 26620226

[28] Milane L, Singh A, Mattheolabakis G, Suresh M, Amiji MM. Exosome mediated communication within the tumor microenvironment. J Control Release. 2015; 219: 278–294. 10.1016/j.jconrel.2015.06.029 26143224

[29] Kharaziha P, Ceder S, Li Q, Panaretakis T. Tumor cell-derived exosomes: a message in a bottle. Biochim Biophys Acta. 2012; 1826: 103–111. 10.1016/j.bbcan.2012.03.006 22503823

[30] Soung YH, Nguyen T, Cao H, Lee J, Chung J. Emerging roles of exosomes in cancer invasion and metastasis. BMB Rep. 2015; in press. 10.5483/BMBRep.2016.49.1.239 PMC4914208 26592936

[31] Kang S, Kim MJ, An H, Kim BG, Choi YP, Kang KS, et al Proteomic molecular portrait of interface zone in breast cancer. J Proteome Res. 2010; 9: 5638–5645. 10.1021/pr1004532 20857901

[32] Xu SG, Yan PJ, Shao ZM. Differential proteomic analysis of a highly metastatic variant of human breast cancer cells using two-dimensional differential gel electrophoresis. J Cancer Res Clin Oncol. 2010; 136: 1545–1556. 10.1007/s00432-010-0812-0 20155427 PMC11828232

[33] Voisin SN, Krakovska O, Matta A, DeSouza LV, Romaschin AD, Colgan TJ, et al Identification of novel molecular targets for endometrial cancer using a drill-down LC-MS/MS approach with iTRAQ. PLoS One. 2011; 6: e16352 10.1371/journal.pone.0016352 21305022 PMC3031560

[34] Sun HQ, Kwiatkowska K, Wooten DC, Yin HL. Effects of CapG overexpression on agonist-induced motility and second messenger generation. J Cell Biol. 1995; 129: 147–156. 7698981 10.1083/jcb.129.1.147 PMC2120377

[35] De Corte V, Van Impe K, Bruyneel E, Boucherie C, Mareel M, Vandekerckhove J, et al Increased importin-beta-dependent nuclear import of the actin modulating protein CapG promotes cell invasion. J Cell Sci. 2004; 117: 5283–5292. 15454578 10.1242/jcs.01410

[36] Zablocki GJ, Ruzycki PA, Overturf MA, Palla S, Reddy GB, Petrash JM. Aldose reductase-mediated induction of epithelium-to-mesenchymal transition (EMT) in lens. Chem Biol Interact. 2011; 191: 351–356. 10.1016/j.cbi.2011.02.005 21329682 PMC3575513

[37] Tammali R, Reddy AB, Srivastava SK, Ramana KV. Inhibition of aldose reductase prevents angiogenesis in vitro and in vivo. Angiogenesis. 2011; 14: 209–221. 10.1007/s10456-011-9206-4 21409599 PMC3103619

[38] Raposo G, Stoorvogel W. Extracellular vesicles: exosomes, microvesicles, and friends. J Cell Biol. 2013; 200: 373–383. 10.1083/jcb.201211138 23420871 PMC3575529

[39] Mathivanan S, Ji H, Simpson RJ. Exosomes: extracellular organelles important in intercellular communication. J Proteomics. 2010; 73: 1907–1920. 10.1016/j.jprot.2010.06.006 20601276

[40] Bazer FW, Ying W, Wang X, Dunlap KA, Zhou B, Johnson GA, et al The many faces of interferon tau. Amino Acids. 2015; 47: 449–460. 10.1007/s00726-014-1905-x 25557050

[41] Yankey SJ, Hicks BA, Carnahan KG, Assiri AM, Sinor SJ, Kodali K, et al Expression of the antiviral protein Mx in peripheral blood mononuclear cells of pregnant and bred, non-pregnant ewes. J Endocrinol. 2001; 170: R7–11. 11479146 10.1677/joe.0.170r007

[42] Shirasuna K, Matsumoto H, Matsuyama S, Kimura K, Bollwein H, Miyamoto A. Possible role of interferon tau on the bovine corpus luteum and neutrophils during the early pregnancy. Reproduction. 2015; 150: 217–225. 10.1530/REP-15-0085 26078085

[43] Oliveira JF, Henkes LE, Ashley RL, Purcell SH, Smirnova NP, Veeramachaneni DN, et al Expression of interferon (IFN)-stimulated genes in extrauterine tissues during early pregnancy in sheep is the consequence of endocrine IFN-tau release from the uterine vein. Endocrinology. 2008; 149: 1252–1259. 18063687 10.1210/en.2007-0863

[44] Romero JJ, Antoniazzi AQ, Nett TM, Ashley RL, Webb BT, Smirnova NP, et al Temporal Release, Paracrine and Endocrine Actions of Ovine Conceptus-Derived Interferon-Tau During Early Pregnancy. Biol Reprod. 2015; 93: 146 10.1095/biolreprod.115.132860 26559679

[45] Caby MP, Lankar D, Vincendeau-Scherrer C, Raposo G, Bonnerot C. Exosomal-like vesicles are present in human blood plasma. Int Immunol. 2005; 17: 879–887. 15908444 10.1093/intimm/dxh267

[46] Berrone E, Corona C, Mazza M, Vallino Costassa E, Lo Faro M, Properzi F, et al Detection of cellular prion protein in exosome derived from ovine plasma. J Gen Virol. 2015; in press. 10.1099/jgv.0.000291 PMC4804764 26399471

